# Assessing accuracy of imputation using different SNP panel densities in a multi-breed sheep population

**DOI:** 10.1186/s12711-016-0244-7

**Published:** 2016-09-23

**Authors:** Ricardo V. Ventura, Stephen P. Miller, Ken G. Dodds, Benoit Auvray, Michael Lee, Matthew Bixley, Shannon M. Clarke, John C. McEwan

**Affiliations:** 1 Centre for Genetic Improvement of Livestock, University of Guelph, Guelph, ON N1G2W1 Canada; 2 Beef Improvement Opportunities, Guelph, ON N1K1E5 Canada; 3 Invermay Agricultural Centre, AgResearch Limited, Mosgiel, 9053 New Zealand; 4 Department of Mathematics and Statistics, University of Otago, Dunedin, 9016 New Zealand

## Abstract

**Background:**

Genotype imputation is a key element of the implementation of genomic selection within the New Zealand sheep industry, but many factors can influence imputation accuracy. Our objective was to provide practical directions on the implementation of imputation strategies in a multi-breed sheep population genotyped with three single nucleotide polymorphism (SNP) panels: 5K, 50K and HD (600K SNPs).

**Results:**

Imputation from 5K to HD was slightly better (0.6 %) than imputation from 5K to 50K. Two-step imputation from 5K to 50K and then from 50K to HD outperformed direct imputation from 5K to HD. A slight loss in imputation accuracy was observed when a large fixed reference population was used compared to a smaller within-breed reference (including all 50K genotypes on animals from different breeds excluding those in the validation set i.e. to be imputed), but only for a few animals across all imputation scenarios from 5K to 50K. However, a major gain in imputation accuracy for a large proportion of animals (purebred and crossbred), justified the use of a fixed and large reference dataset for all situations. This study also investigated the loss in imputation accuracy specifically for SNPs located at the ends of each chromosome, and showed that only chromosome 26 had an overall imputation (5K to 50K) accuracy for 100 SNPs at each end higher than 60 % (r^2^). Most of the chromosomes displayed reduced imputation accuracy at least at one of their ends. Prediction of imputation accuracy based on the relatedness of low-density genotypes to those of the reference dataset, before imputation (without running an imputation software) was also investigated. FIMPUTE V2.2 outperformed BEAGLE 3.3.2 across all imputation scenarios.

**Conclusions:**

Imputation accuracy in sheep breeds can be improved by following a set of recommendations on SNP panels, software, strategies of imputation (one- or two-step imputation), and choice of the animals to be genotyped using both high- and low-density SNP panels. We present a method that predicts imputation accuracy for individual animals at the low-density level, before running imputation, which can be used to restrict genomic prediction only to the animals that can be imputed with sufficient accuracy.

## Background

Imputation refers to a statistical approach that is able to infer single nucleotide polymorphism (SNP) genotypes, which are not obtained from a low-density panel, by using information from a group of animals that are genotyped with higher density panels [1–3]. Widespread implementation of genomic selection [4] in dairy cattle quickly followed the development of the Illumina SNP50 Genotyping beadchip [5]. The technology was subsequently launched for sheep [6] and beef cattle [7] as reference datasets of genotyped animals with a suitable size became available, as well as SNP panels (http://support.illumina.com/array/array_kits/). The next advancement in the technology was the use of lower density panels, which are available at a lower cost compared to the higher density panels required for genomic selection, and can be imputed to higher densities with high accuracy in cattle [1, 8–10]. Imputation is also a key strategy for the implementation of genomic selection within the New Zealand sheep industries [6].

Several studies have investigated accuracy of genotype imputation and its impact on the accuracy of genomic selection in dairy and beef cattle through the adoption of high-density SNP panels, and more recently, whole-sequence data [1, 11–17]. Several panels that vary in the number of SNPs they include are currently available on the market and the number of genotyped individuals is rapidly growing in livestock sectors due to the reduction in costs and the development of new genotyping tools [9]. Although the imputation efficiency of each SNP panel is well documented [1, 18, 19], few articles evaluated imputation accuracy across different panels using both crossbred and purebred populations [20, 21] and, more specifically, strategies for the prediction of imputation accuracy are scarce.

Imputation is a robust tool to minimize costs of genotyping, but many factors can influence imputation accuracy, which provide opportunities for further improvements and optimal implementation of this technology. For some animal populations, missing SNPs cannot be inferred with high accuracy and this depends on the structure of the reference population (i.e. the group of animals genotyped with high-density SNPs) and the marker density of both reference and imputed populations. Gains in imputation accuracy are closely associated with the level of relationship between the animals to be imputed and the reference population, the number of animals in the reference population, the position of the SNPs on the chromosome, the density of the SNP panel used for the reference population, and the breed composition [1, 9, 13, 22].

Imputation of rare alleles is a particularly difficult task that is directly associated with minor allele frequencies (MAF); it can influence accuracy of genomic selection because of the potential influence of such alleles on the genetic expression of the trait under study [9, 23]. For example, for a chromosomal region that contains SNPs with a low MAF, association methods can generate spurious results due to genotyping errors [24]. Variants with a MAF lower than 0.05 could be under selection or in a related process that removes them from the population. According to Sargolzaei et al. [9], such variants with a low MAF tend to be recent mutations and are more likely to be identified after detecting long haplotypes. The same study [9] reported gains in imputation accuracy by using information on relatives, which can also optimize the imputation of rare alleles compared with other algorithms. Different measures of accuracy have been implemented, which depend on the methods used to compare the original and imputed genotypes, and the output generated from each software/method [12, 15]. Calus et al. [13] evaluated different measures of correctness of genotype imputation in the context of genomic prediction and suggested that correlation between imputed and true genotypes is the most useful and unbiased measure of imputation accuracy and is suitable for comparisons across loci regardless of the MAF of SNPs [13]. The same authors suggested that individual specific imputation accuracies should be computed from genotypes that are centered and scaled. We did not apply this approach in our investigation but plan to evaluate it in future studies.

Hayes et al. [14] evaluated the accuracy of genotype imputation from low-density to 50K panels in sheep breeds by comparing fastPHASE [25] and BEAGLE [26] software programs. Recently, a new approach for efficient genotype imputation was reported by Sargolzaei et al. [9] and is implemented in the newest version of the FIMPUTE software. Ventura et al. [1] assessed the impact of the reference population on accuracy of imputation from 6K and 50K SNP chips in purebred and crossbred beef cattle. These authors showed that IMPUTE2 and FIMPUTE imputed almost all the individuals more accurately than BEAGLE by testing several scenarios and that they were also very efficient in terms of run time.

The objective of our study was to provide practical directions on the implementation of imputation strategies in a multi-breed sheep population that was genotyped with three SNP panels: 5K, 50K and HD (600K SNPs), and to compare these strategies with the current implementation of imputation that is carried out in practice for genomic selection in the New Zealand sheep industry. We evaluated: (1) composition of the reference population; (2) SNP density; (3) imputation of rare variants; (4) imputation software; (5) measures of imputation accuracy; and (6) prediction of imputation accuracy.

## Methods

Population imputation was implemented using BEAGLE 3.3.2 [26] and FIMPUTE 2.2 software [9] and several scenarios were generated by alternating the animals that were included in the reference population and in the set of animals to be imputed. The reference population consisted of animals that were genotyped with the Illumina OvineSNP50 Genotyping BeadChip (53,903 SNPs) (http://www.illumina.com/products/ovinesnp50_dna_analysis_kit.html) and/or the Ovine Infinium^®^ HD SNP BeadChip (603,350 SNPs). Only autosomal SNPs were included in this study.

### Data

**Fig. 1:**
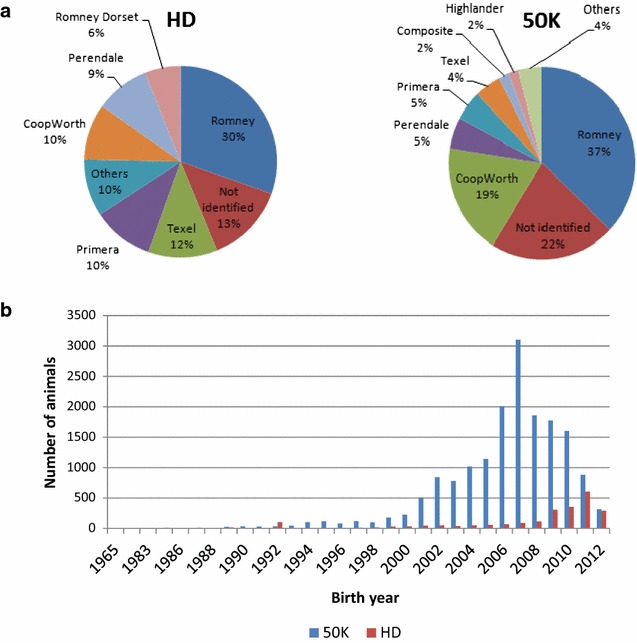
Distribution of animals genotyped with 50K and HD. According to **a** main breed composition and **b** birth year

A dataset including 2409 animals that were genotyped with the Ovine Infinium^®^ HD and 17,176 animals that were genotyped with the Illumina OvineSNP50 were used to evaluate imputation accuracy. Before describing the imputation scenarios that were used to evaluate issues such as relatedness, multi- versus one-breed reference population and SNP density, we present the multi-breed populations according to the density of the SNP panel used to genotype animals and to the proportion of the main breed that composes the population. Animals in this dataset were primarily sires from breeders’ flocks along with a group of animals of both sexes from research flocks. Average breed composition as deduced from pedigree information is described here for the two groups of animals that were genotyped with the 50K and HD panels:

1. 50K animals: 37 % Romney (30 % purebred Romney), 19 % Coopworth (8 % purebred), 4 % Texel (1 % purebred), 6 % Perendale (5 % purebred), 5 % Primera (composite of terminal sire breeds http://www.focusgenetics.com/sheep/sheep-breeding-programme/primera/) and other breeds with less than 3 % each.
2. HD animals: 33 % Romney (30 % purebred Romney), 10 % Coopworth (7 % purebred), 12 % Texel (1 % purebred), 9 % Perendale (6 % purebred), 11 % Primera (8 % purebred) and for the remaining animals, the breed was not identified (this set of individuals was not incorporated in any of our imputation scenarios). The distribution of the animals per breed/group is in Fig. 1a. This information was used to guide the choice of the most suitable imputation scenario since it is mainly influenced by factors such as number of breeds/groups available for investigation and number of individuals genotyped at each density. Animals that were genotyped with the HD panel but with an unknown breed composition were excluded from our investigation since they were not connected with the groups of animals analyzed, as determined by cluster analysis. The distribution of the genotyped animals for each panel density (50K or HD) according to birth year is in Fig. 1b.

### Genotype conversion and quality control

Animals were genotyped with the Illumina OvineSNP50 and the Ovine Infinium^®^ HD panels. Genotypes were coded as 0, 1, or 2 for AA, AB and BB genotypes, A and B being the two alleles of an SNP. Quality controls included removal of SNPs that (1) did not have defined positions on the ovine genome, (2) had a minor allele frequency (MAF) lower than 0.0005, (3) had a call rate lower than 95 % or (4) deviated from Hardy–Weinberg equilibrium (threshold p value: 1 × 10^−5^). Finally, 48,241 and 568,569 autosomal SNPs (from the original 50K and HD panels, respectively) were retained for the analyses. In addition, genotyped animals were excluded if their average genotype call rate was lower than 95 %.

### Design of the low-density SNP panel

Two low-density SNP panels (5K and 50K) were simulated to test imputation by deleting part of the SNPs from the 50K and HD panels, i.e.:

1. only SNPs that were shared between the Illumina Ovine 5K SNP chip (http://www.illumina.com/documents/products/datasheets/) that is used commercially for genomic selection in New Zealand sheep [27] and the 50K original panel were retained, which resulted in 5095 SNPs (5K)
2. only SNPs that were shared between the Illumina OvineSNP50 and the Ovine Infinium^®^ HD panels were retained, which resulted in 41,708 SNPs (50K).

### Genomic relationships between animals from different breeds were determined by clustering

Relatedness is one of the key factors that affect the success of any imputation process. The genomic relationship matrix (**G** matrix) was calculated as follows and used for clustering analysis to verify the genetic connectivity (based on SNPs) among individuals from different breeds. In order to verify the connection of the genotyped animals among different breeds/groups and to better define the imputation scenarios, 100 animals from each breed or group were randomly selected to derive the **G** matrix and a cluster analysis was implemented by using the multidimensional scaling (MDS) approach, which is part of the package ggplot2 in R language. The **G** matrix was calculated as:

$${\mathbf{G}} = \frac{{{\mathbf{XX}}'}}{{2\sum p_{i} \left( {1 - p_{i} } \right)}}\;\left[ {28} \right],$$

where *p*_*i*_ is the allele frequency of the *i*-*th* SNP and **X** is the incidence matrix for SNPs.

### Imputation scenarios

Thirty-one imputation scenarios were considered and animals in the reference population were selected based on the following criteria: density of the SNP panel (50K or HD), birth year (older animals), breed composition (multi- versus one-breed) and level of genomic relationship with imputed animals, as described in Tables 1, 2 and 3. For most of the 31 scenarios, the set of animals with imputed genotypes was composed of younger animals, which had their HD or 50K genotypes masked back to 50K or 5K genotypes, respectively.

**Table 1:** Imputation scenarios with HD genotypes using different groups of purebred and crossbred animals

Scenario^a^	Number of reference animals^b^	Number of imputed animals	Description of reference animals	Density of reference panel^c^	Imputed group breed^d^	Density of panel of imputed animals
1_5K50K	500	116	Romney	50K	Romney	5K
1B_5KHD_1STEP	500	116	Romney	HD	Romney	5K
1B_5KHD_2STEP	17,000 + 500	116	Romney	HD	Romney	5K
2_50KHD	500	116	Romney	HD	Romney	50K
3_5K50K	469	116	Romney-31 animals related with the imputed group	50K	Romney	5K
3B_5KHD	469	116	Romney-31 animals related with the imputed group	HD	Romney	5K
4_50KHD	469	116	Romney-31 animals related with the imputed group	HD	Romney	50K
5_5K50K	500 (R) + 100 (P)	116	Romney + Perendale	50K	Romney	5K
5B_5KHD	500 (R) + 100 (P)	116	Romney + Perendale	HD	Romney	5K
6_50KHD	500 (R) + 100 (P)	116	Romney + Perendale	HD	Romney	50K

^a^Imputation scenarios were from 5K to 50K (50K was a subset of the HD panel), 5K to HD and 50K to HD

^b^2-Step imputation: from 5K to 50K using all genotyped animals as reference population (N = 17,000) and from 50K imputed to HD using 500 animals as the reference population

^c^The oldest animals in each scenario were used as reference population

^d^The youngest animals in each scenario were imputed

**Table 2:** Imputation scenarios with 50K genotypes using different groups of purebred and crossbred animals

Scenario^a^	Number of reference animals^b^	Number of imputed animals	Description of reference animals	Imputed group breed^c^
7_5K50K	466	500	Romney	Romney
8_5K50K	933	500	Romney	Romney
9_5K50K	1860	500	Romney	Romney
10_5K50K	2860	500	Romney	Romney
11_5K50K	4862	500	Romney	Romney
12_5K50K	933	200	Romney	Composite
13_5K50K	1000 (R) + 893 (C)	200	Romney + Coopworth	Composite
14_5K50K	1000 (R) + 893 (C) + 500 (P) + 500 (T)	200	Romney + Coopworth + Perendale + Texel	Composite
15_5K50K	710	500	Primera	Romney
16_5K50K	710 (P) + 933 (R)	500	Primera + Romney Scenario 8	Romney
17_5K50K	710 (P) + 1860 (R)	500	Primera + Romney Scenario 9	Romney
18_5K50K	350	200	Primera	Primera
19_5K50K	506	200	Primera	Primera
20_5K50K	350 (P) + 77 (S,PD)	200	Primera + Suffolk + Poll Dorset	Primera
21_5K50K	506 (P) + 77 (S,PD)	200	Primera + Suffolk + Poll Dorset	Primera
22_5K50K	470	300	Coopworth	Coopworth
23_5K50K	951	300	Coopworth	Coopworth
24_5K50K	951 (C) + 933 (R)	300	Coopworth + Romney	Coopworth

^a^Imputation scenarios were from 5K to 50K (original 50K panel)

^b^The oldest animals in each scenario were used as the reference population

^c^The youngest animals in each scenario were imputed

**Table 3:** Imputation scenarios from 5K to 50K (50K original) using two types of reference population

Scenario	Number of reference animals	Number of imputed animals	Description of reference animals^d^	Imputed group breed^b^
25_5K50K	15,443^a^ and 4564^b^	218	All breeds/Romney	Romney 100 %
26_5K50K	15,443^a^ and 4326^b^	142	All breeds/Romney	Romney < 65 %
27_5K50K	4256	1000^c^	Romney	Romney
28_5K50K	15,443^a^ and 2324^b^	250	All breeds/Coopworth	Coopworth 100 %
29_5K50K	15,443^a^ and 2279^b^	250	All breeds/Coopworth	Coopworth < 70 %
30_5K50K	15,443^a^ and 640^b^	250	All breeds/Perendale	Perendale > 95 %
31_5K50K	15,443^a^ and 138^b^	172	All breeds/Composites	Composites > 50 % < 95 %

^a^Fixed reference population that included 15,443 animals from all breeds with genotyped animals

^b^Within-breed/group reference population: some groups contained a small number of genotyped animals

^c^1000 animals defined as the imputed set to optimize the calculation of the r^2^ imputation accuracy per SNP

^d^Two types of reference population were used: (1) a fixed reference population that included a large number of animals from all breeds and (2) a within-group reference population

The ten scenarios that are listed in Table 1 were designed to investigate different SNP densities and imputation of purebred Romney animals using alternate reference sets. These scenarios consisted in the imputation of the 116 youngest Romney animals using the oldest 500 Romney animals as reference population (except for the 2STEP Scenario, which included 17,000 animals that were genotyped with the 50K panel and constituted the reference set during the first step of imputation from 5K to 50K).

### One-step versus two-step with a larger intermediate density reference set

In Scenario 1B_5KHD_2STEP, imputation from 5K to HD was done by using a two-step approach: from 5K to 50K and then from 50K to HD. This scenario allowed us to determine if a larger dataset that included animals genotyped with the 50K panel would improve haplotype reconstruction and hence imputation accuracy.

### Relatedness, and impact of the size and breed composition of the reference population

In Scenarios 3, 3B and 4, 31 animals were excluded from the reference population because their relationship with at least one animal from the group of animals with imputed genotypes resulted in a relationship coefficient (based on the **G** matrix) that was higher than 0.45 (defined after parentage testing). In Scenarios 5, 5B and 6, randomly selected animals from another related breed (Perendale) were added to the reference population.

The scenarios that are listed in Table 2 evaluated the efficiency of imputation from 5K to 50K for Romney, composite, Primera terminal composite group (http://www.focusgenetics.com/sheep/sheep-breeding-programme/primera) and Coopworth animals (genotypes were obtained with the 50K Illumina panel and not with a subset from the HD panel). Combining 50K genotypes and subsets of genotypes obtained with the HD panel resulted in a larger number of animals available for the analyses.

For Scenarios 7_5K50K to 11_5K50K, within-breed imputation of 510 Romney animals was performed by enlarging the reference set (n = 466, 933, 1860, 2862 and 4862, respectively), i.e. by sorting the animals according to birth year and then by selecting them randomly within year groups.

For Scenarios 15_5K50K to 17_5K50K, the Primera set was first used as reference population (N = 710) to impute Romney animals (N = 500, Scenario 15_5K50K). Scenarios 16_5K50K and 17_5K50K were performed to check the effect of including Romney animals (same group of animals as in Scenarios 8_5K50K and 9_5K50K) to compose a multi-breed reference population. Scenarios 18_5K50K to 21_5K50K were used to evaluate the imputation of Primera animals (N = 200) by enlarging the reference population (N = 350 and 506) and combining animals from breeds that were used to create the Primera terminal composite group (Suffolk and Poll Dorset, N = 77). The last three scenarios in Table 2 (Scenarios 22_5K50K, 23_5K50K and 24_5K50K) aimed at investigating the imputation of Coopworth animals (N = 300) after doubling the size of the reference population (from 470 to 951, Scenarios 22_5K50K and 23_5K50K, respectively) and the impact of adding Romney animals in the reference population (Scenario 24_5K50K, N = 934).

### Imputation of composite animals by expanding related breeds in the reference population

Scenarios 12_5K50K, 13_5K50K and 14_5K50K were used to evaluate imputation of composite animals by (1) using only Romney animals in the reference population (Scenario 12_5K50K), (2) adding Coopworth animals (Scenario 13_5K50K), and (3) including Perendale and Texel animals in the reference population (Scenario 14_5K50K). In New Zealand, much of the genetic background of commercial ewes used as dual-purpose sheep as studied here, originates from the Romney breed and both the Coopworth and Perendale breeds have a Romney origin. Texel is a breed that has recently been used in composite dual-purpose meat sheep to increase lean yield [6, 27].

### Within-group imputation or use of a fixed reference population that includes animals from all breeds with HD genotypes

Table 3 describes Scenarios 25_5K50K to 31_5K50K that aimed at assessing imputation accuracy of Romney (25_5K50K and 26_5K50K), Coopworth (28_5K50K and 29_5K50K), Perendale (30_5K50K) and composite (31_5K50K) animals; two different reference populations were used for each scenario: (1) a fixed reference population that included a large group of animals from all breeds (N = 15,443) and (2) a within-breed reference population. Romney and Coopworth imputed animals were also divided into two subgroups each, according to breed proportion: 100 % Romney or < 65 % (Scenarios 25_5K50K and 26_5K50K, respectively) and 100 % or < 70 % Coopworth (Scenarios 28_5K50K and 29_5K50K, respectively.

### Imputation of rare alleles and accuracy of imputation for SNPs located at the ends of chromosomes

Scenario 27_5K50K was specifically designed to investigate within-breed imputation of Romney animals for rare alleles and to verify regions with reduced imputation accuracy using the squared Pearson correlation coefficient as a measure of accuracy. This scenario had the largest number of imputed animals and was deemed best to test imputation accuracy of rare variants.

### Prediction of imputation accuracy before imputing missing genotypes

Based on SNP data, the relatedness among animals from the imputed and reference populations was investigated for each scenario, as the genomic relationship average value (extracted from the **G** matrix) between each imputed animal and the 10 most related individuals from the reference population. The minimum and maximum top 10 relationships (upper and lower value for each group of the 10 most related animals) for each scenario were also calculated to compare the estimated accuracies of imputation. Another measure of relatedness was also investigated to predict imputation accuracy before running the imputation process: Mendelian inconsistency (MI), which is the average number of Mendelian inconsistencies between an imputed animal and the top 10 related individuals from the reference group, where MI reflects the number of opposing homozygotes between two individuals. Two individuals that have high MI values after genotype comparison are likely to share fewer haplotypes than individuals that have a low MI value.

### Comparison of imputation software packages

We compared two software packages: BEAGLE and FIMPUTE. We do acknowledge that changes to BEAGLE software are now available (Version 4) and that this new version should be evaluated in future studies, along with any other available updates of these software packages, to determine if there are advantages for the New Zealand sheep industry. BEAGLE exploits linkage disequilibrium between SNPs and implements a population imputation method that assumes that all animals are unrelated. This software uses a hidden Markov model (HMM) and a localized haplotype clustering method to infer genotypes as described by Browning et al. [26]. All analyses using BEAGLE were carried out by setting default parameters. The FIMPUTE software uses a deterministic approach that combines family and population imputation methods. The population imputation method is based on the assumption that all individuals have some degree of relationship and share haplotypes that may differ in frequency and length depending on the relationships. FIMPUTE is a two-step procedure, i.e. first it searches for long haplotypes by applying a family imputation method, and second, it identifies short segments (two SNPs) by applying a population imputation method that analyzes overlapping sliding windows. BEAGLE analyses that were not complete within 1 week of computing time or failed at least twice during the process (the cause of failure could not be determined) were excluded and are not presented in this paper (13 occurrences).

### Determination of imputation accuracy

Imputation accuracy (per individual and per SNP) was determined with two different measurements: (1) allelic squared Pearson correlation coefficient (r^2^) as an appropriate approach to minimize the dependency on allele frequency and (2) concordance rate: proportion of correctly called SNP genotypes versus all called SNPs. Both values were determined by comparing imputed and true genotypes. Since imputation accuracy of specific SNPs was useful for Scenario 27_5K50K, which investigated imputation of rare variants, r^2^ per SNP was calculated.

### Run-time comparison (overall computing time)

FIMPUTE analyzes a set of chromosomes simultaneously by implementing parallel computing. For each software package, the total length of running time (overall computing time) was measured for all scenarios but comparison of values between BEAGLE 3.3.2 and FIMPUTE was not possible. Due to the long computation time required with BEAGLE, these analyses were carried out using the Condor server located at the University of Wisconsin (Linux server (fedora core 16) with dual Intel Xeon X5690@3.47 GHz CPUs). FIMPUTE analyses were performed on a local server, located at the Invermay Agricultural Centre, Agresearch (Linux server (CentOS 6.5) with 48 AMD Opteron 6176SE @2.3 GHz CPUs). Ten parallel jobs were implemented for BEAGLE and FIMPUTE, for comparison among scenarios (within software).

## Results

**Fig. 2:**
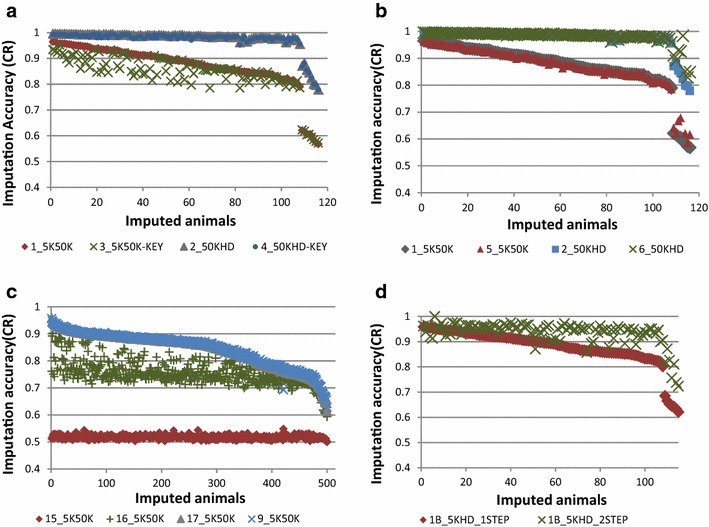
Imputation accuracy assessed by alternative approaches. **a** Imputation from 5K to 50K (Scenarios 1_5K50K and 3_5K50K) and from 50K to HD (Scenarios 2_50KHD and 4_50KHD) using the FIMPUTE software for purebred Romney animals (The suffix “KEY” refers to the 31 animals that are highly related with the group of imputed animals). **b** Imputation from 5K to 50K (Scenarios 1_5K50K and 5_5K50K) and from 50K to HD (Scenarios 2_50KHD and 6_50KHD) using the FIMPUTE software for purebred Romney animals after including Perendale animals in the reference set. **c** Imputation from 5K to 50K using the FIMPUTE software for purebred Romney animals after using the Primera group as reference set (Scenario 15_5K50K) and inclusion of Romney animals in the reference set (Scenarios 16_5K50K and 17_5K50K). Scenario 9 was included in this plot for comparison with within-breed imputation. **d** Imputation from 5 K to HD using the FIMPUTE software for purebred Romney animals by one- or two-step imputation (Scenarios 1B_5KHD_1STEP and 1B_5KHD_2STEP). The *x*-axis represents the number of imputed individuals sorted from the highest to the lowest accuracy value

**Fig. 3:**
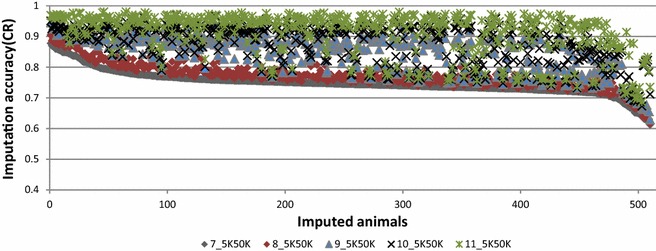
Imputation from 5K to 50K using the FIMPUTE software for purebred Romney animals. Scenarios 7_5K50K to 11_5K50K. The *x*-axis represents the number of imputed individuals sorted from the highest to the lowest accuracy value

**Fig. 4:**
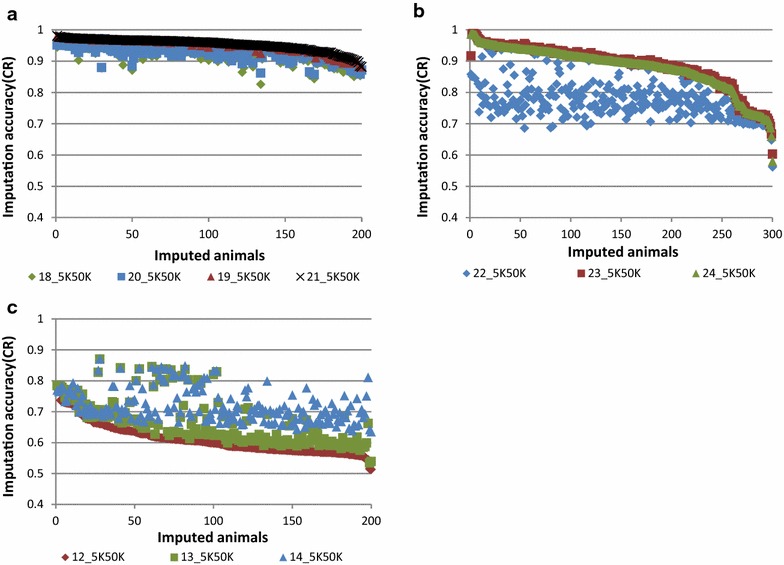
Imputation accuracy combining different breeds. **a** Imputation from 5K to 50K (Scenarios 18_5K50K to 21_5K50K) using the FIMPUTE software for purebred Primera animals after enlarging the reference set within group or adding animals from other breeds. **b** Imputation from 5K to 50K (Scenarios 22_5K50K to 24_5K50K) using the FIMPUTE software for Coopworth animals after enlarging the reference set within group or adding Romney animals. **c** Imputation of composite animals using alternate sets of reference population from 5K to 50K using the FIMPUTE software (Scenarios 12_5K50K to 14_5K50K). The *x*-axis represents the number of imputed individuals sorted from the highest to the lowest accuracy value

**Fig. 5:**
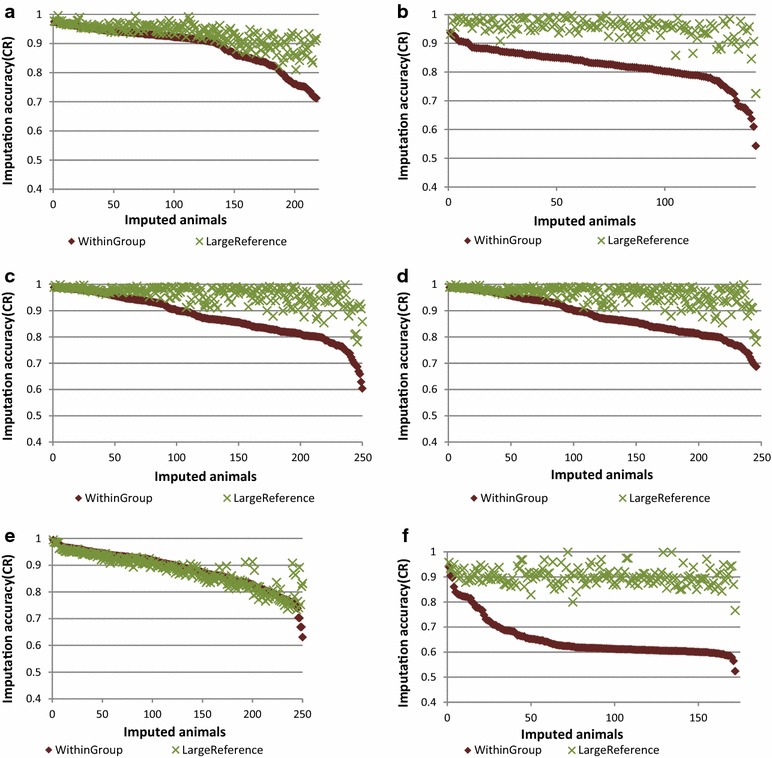
Imputation accuracy using large fixed or within-group reference populations. Imputation from 5K to 50K (Scenarios 25–31) using the FIMPUTE software under different scenarios and two types of reference population: (i) fixed reference population containing a large number of animals from all breeds and (ii) within-group reference population. The *x*-axis represents the number of imputed individuals sorted from the highest to the lowest accuracy value. **a** Scenario 25, **b** Scenario 26, **c** Scenario 28, **d** Scenario 29, **e** Scenario 30, **f** Scenario 31

**Fig. 6:**
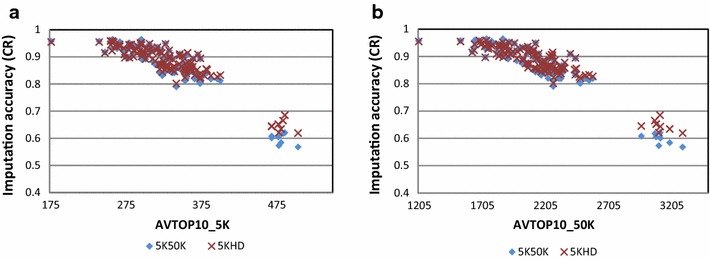
Imputation accuracy and its relation with the connectivity between each imputed animal and the reference set. Average numbers of Mendelian inconsistencies (AVTOP10_5K and AVTOP10_50K) between each animal in the imputed set and all animals from the reference set were calculated and are presented for each imputed animal as the average of 10 pairs of animals (one from the reference set and one from the imputed set) with the lowest Mendelian inconsistency. Imputation from 5K to 50K (Scenario 1_5K50K) and 5K to HD (Scenario 1B_5KHD_1STEP) using the FIMPUTE software for purebred Romney animals is also compared with the value defined above. **a** AVTOP10_5K calculated using the 5K panel before imputation. **b** AVTOP10_50K calculated using the 50K panel. The *x*-axis represents the average number of Mendelian inconsistencies and the *y*-axis the imputation accuracy per animal measured by concordance rate (CR)

**Fig. 7:**
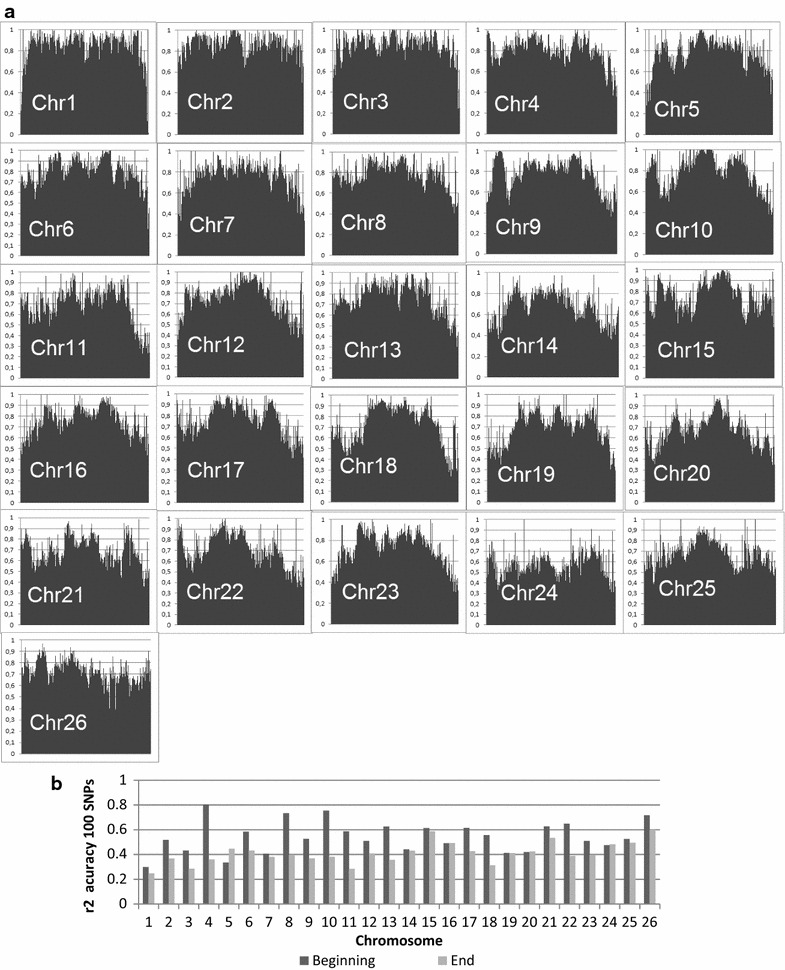
Imputation accuracy per chromosome and at both chromosome ends. **a** Squared Pearson correlation measure of imputation accuracy (r^2^) across different chromosomes after imputation from 5K to 50K for Romney sheep using the FIMPUTE software (Scenario 27_5K50K). **b** Squared Pearson correlation measure of imputation accuracy (r^2^) for both ends of each chromosome (each chromosome end is covered by 100 markers); imputation accuracy defined as the average r^2^ value for the 100 markers

In this paper, tables are used to report the concordance rate (CR) and r^2^ measures of imputation accuracy, and figures show the variation in imputation efficiency for all animals genotyped with the low-density panel in each scenario. All figures provide imputation accuracy per animal in terms of CR. Tables 4, 5, 6, and 7 and Figs. 2, 3, 4, 5, 6 and 7 report the results for Scenarios 1–31 that are defined in Tables 1, 2 and 3.

**Table 4:** Accuracy of genotype imputation and computing time for BEAGLE and FIMPUTE algorithms

Scenario	CR_F^a^	r^2^_F^b^	Run Time_F m:s	CR_B^c^	r^2^_B^d^	Run Time_ B h:m:s	Mean Top10^e^	Min Top10^e^	Max Top10^e^
1_5K50K	86.98	78.75	00:57	83.80	73.80	02:16:25	0.115	0.034	0.234
1B_5KHD_1STEP	87.61	80.73	06:51	84.10	74.00	23:12:35	0.115	0.034	0.234
1B_5KHD_2STEP	93.28	89.6	–	NA	NA	NA	0.115	0.034	0.234
2_50KHD	97.56	96.2	07:42	96.98	95.42	21:55:35	0.115	0.034	0.234
3_5K50K	84.35	74.15	00:53	82.12	70.94	03:15:10	0.090	0.033	0.179
3B_5KHD	85.3	76.85	06:43	82.23	71.12	27:17:35	0.090	0.033	0.179
4_50KHD	97.25	95.71	07:11	96.63	94.91	12:33:02	0.090	0.033	0.179
5_5K50K	87.19	78.98	01:08	83.58	73.37	03:18:52	0.097	0.037	0.252
5B_5KHD	87.68	80.81	08:45	83.99	76.00	25:16:22	0.097	0.037	0.252
6_50KHD	98.06	97.01	09:14	Failed	Failed	Failed	0.097	0.037	0.252

^a^CR_F = concordance rate using the FIMPUTE software

^b^r^2^_F = Squared Pearson correlation using the FIMPUTE software

^c^CR_B = concordance rate using the BEAGLE software

^d^r^2^_B = Squared Pearson correlation using the BEAGLE software

^e^Mean Top10, Min Top10 and Max Top10 = mean, min and max relationship among the 10 most related animals between the reference and imputed sets

First, we assessed imputation accuracy using two population imputation methods (BEAGLE and FIMPUTE) applied to HD genotypes of purebred Romney animals.

### One- versus two-step imputation

The two-step imputation scenario (Scenario 1B_5KHD_2STEP) that imputed animals first from 5K to 50K and then, from 50K imputed to HD, was compared to the one-step imputation scenario from 5K to HD (Scenario 1B_5KHD_1STEP), which showed that the two-step procedure increased imputation accuracy by 5.67 % (CR) and 8.87 % (r^2^). Based on Fig. 2d, animals for which imputation accuracy (CR) was lower than 95.1 % using the one-step approach, inference of missing genotypes was more efficient with the two-step procedure.

Imputation from a medium-density panel (50K) to HD (Scenarios 2_50KHD, 4_50KHD and 6_50KHD) resulted in the highest imputation accuracies i.e. higher than 97.25 % (CR) (see Table 4).

### Imputation from 5K to both 50K and HD panels using one or more breeds in the reference population and impact of relatedness on imputation accuracy

Table 4 shows the accuracy of genotype imputation from 5K to 50K and HD, and from 5K to 50K. All SNP panels represented a subset of the HD panel. The highest CR (87.19 %) and r^2^ (78.98 %) values (Table 4) for imputation from 5K to 50K were obtained when Romney and Perendale animals were combined in the reference population (Scenario 5_5K50K; Table 1). The difference in overall average imputation accuracy between Scenarios 1_5K50K and 5_5K50K was very small (0.21 and 0.23 % for CR and r^2^, respectively). Figure 2b shows that a small improvement in imputation accuracy for imputation to 50K and HD was observed for some animals for which CR accuracy was lower than 70 % (imputation to 50K) in Scenario 1_5K50K and imputation of genotypes was improved by adding Perendale animals in the training dataset.

On average, CR accuracy and r^2^ decreased by 2.63 and 4.60 %, respectively, when 31 Romney animals, which were highly related with the animals that had imputed genotypes, were removed from the training dataset (comparison of Scenarios 1_5K50K and 3_5K50K). As also shown by Fig. 2a, the removal of these 31 animals caused a decrease in imputation accuracy for imputation from low-density to 50K for several animals in all ranges of accuracy, except for the seven animals that showed the lowest imputation efficiencies (CR < 70 %). For this set of animals, average MI values were higher than 3000, which indicates a low level of relationship compared to the animals in the reference population. Imputation from 50K to HD (Scenario 4_50KHD) was not affected by removing the key related animals from the reference population. Imputation from 5K to HD (3B_5KHD) was also performed in this study and imputation accuracies (CR using FIMPUTE) were on average slightly higher (0.95 %) than for imputation from 5K to 50K (3_5K50K). Imputation accuracy from 5K to HD ranged from 82.23 to 87.68 % (CR) and from 71.12 to 80.81 % (r^2^) for Scenarios 1B_5KHD, 3B_5KHD and 5B_5KHD (see Table 4).

### Size of the reference set

The first five scenarios (Scenarios 7_5K50K to 11_5K50K) were used to evaluate the within-breed accuracy of imputation for 510 Romney animals by enlarging the reference population from 466 to almost 5000 animals. CR (and r^2^) accuracies ranged from 74.82 % (57.79 %) for Scenario 7_5K50K to 91.06 % (85.38 %) for Scenario 11_5K50K, respectively. The highest accuracy was reached when 4862 animals (the largest set of Romney animals) were included in the reference population (Scenario 11_5K50K). Figure 3 shows imputation accuracy (CR) per animal for the same set of results presented above. A large average gain in accuracy (16.24 %) was obtained by increasing the reference population by tenfold.

### Imputation of composite animals, multi- versus one-breed reference population and use of a single reference population for all imputed animals

The overall average imputation accuracy of composite animals using different reference populations that consisted of Romney animals and additional individuals from other groups (Coopworth, Perendale and Texel) ranged from 60.93 to 72.12 % (CR) and from 35.25 to 52.44 % (r^2^) (Scenarios 12_5K50K to 14_5K50K). As shown in Fig. 4c, gains in imputation accuracy per animal were obtained by adding animals from different breeds to the reference population.

Accuracies of imputation of Romney animals using a reference population that comprised animals from another breed (Primera) were close to those of imputation by chance (i.e. replacing a missing genotype by the allele of higher frequency), also defined as random imputation (CR = 51.82 % and r^2^ = 17.89 % (Scenarios 15_5K50K to 17_5K50K). Addition of Romney animals to the reference population (Scenarios 16_5K50K and 17_5K50K) increased imputation accuracy to values that were similar to those obtained for within-breed imputation (Scenario 9_5K50K; Fig. 2c). Overall, average gains in accuracy of 2.89 % in CR and 5.12 % in r^2^ were observed by enlarging the reference Primera population (Scenarios 18_5K50K and 19_5K50K) with animals related to those that were at the origin of this group (Suffolk and Poll Dorset) (0.22 % in CR and 0.36 % in r^2^). Only 6 % of the animals from the imputed set showed little overall gain in accuracy (2.3 %) by including animals from the two additional breeds (Fig. 4a). A slight decrease (0.47 % in CR and 0.78 % in r^2^) in imputation accuracy was observed when Romney animals were included in the scenario for which Coopworth individuals were used in both the reference and imputed sets (Scenarios 23_5K50K to 24_5K50K). A near two-fold reduction in reference population size decreased imputation accuracy more than the addition of a second breed in the reference population, which resulted in a very slight decrease in accuracy (Fig. 4b). With FIMPUTE software and across all scenarios (see Table 5), the shortest and longest computing times were observed for Scenario 18_5K50K (44 s) and Scenario 9_5K50K (5 min and 47 s), respectively.

**Table 5:** Accuracy of genotype imputation from 5K to 50K and computing time when using the FIMPUTE software

Scenario	CR_F^a^	r^2^_F^b^	Run time_F m:s	Mean Top10^c^	Min Top10^c^	Max Top10^c^
7_5K50K	74.82	57.79	01:15	0.058	0.011	0.178
8_5K50K	77.10	61.64	02:14	0.076	0.036	0.210
9_5K50K	84.42	74.05	03:33	0.135	0.054	0.310
10_5K50K	87.55	79.29	05:47	0.152	0.052	0.394
11_5K50K	91.06	85.38	09:08	0.177	0.054	0.398
12_5K50K	60.93	35.25	02:04	0.085	0.055	0.168
13_5K50K	66.69	44.25	03:32	0.095	0.056	0.338
14_5K50K	72.12	52.44	05:47	0.123	0.056	0.349
15_5K50K	51.82	17.89	01:28	0.004	0.003	0.006
16_5K50K	75.18	58.25	03:07	0.117	0.052	0.259
17_5K50K	84.07	73.41	05:04	0.153	0.058	0.335
18_5K50K	92.21	86.78	00:44	0.140	0.091	0.183
19_5K50K	95.10	91.90	01:01	0.042	0.001	0.270
20_5K50K	92.8	87.77	00:55	0.045	0.001	0.187
21_5K50K	95.32	92.26	01:11	0.066	0.002	0.270
22_5K50K	77.53	62.36	01:07	0.070	0.022	0.211
23_5K50K	88.46	80.92	02:05	0.167	0.023	0.370
24_5K50K	87.99	80.14	03:34	0.204	0.055	0.417

^a^CR_F = concordance rate when using the FIMPUTE software

^b^r^2^_F = Squared Pearson correlation when using the FIMPUTE software

^c^Mean Top10, Min Top10 and Max Top10 = mean, min and max relationship among the 10 most related animals between the reference and imputed sets

Overall, average gains in accuracy of 8.52 % in CR and 14.03 % in r^2^ were obtained for all scenarios that compared a within-group reference population versus a fixed and large reference population that comprised animals from all groups (Table 6, Scenarios 25_5K50K to 31_5K50K).

**Table 6:** Accuracy of genotype imputation with the FIMPUTE software using two types of reference population

Scenario^a^	CRAll^c^	r^2^All^c^	CRW^d^	r^2^W^d^	MeanA^e^	MinA^e^	MaxA^e^	MeanW^e^	MinW^e^	MaxW^e^
25_5K50K	93.39	89.38	89.16	82.17	0.023	0.079	0.467	0.145	0.049	0.376
26_5K50K	95.45	92.10	82.05	70.47	0.267	0.096	0.432	0.185	0.077	0.355
27_5K50K^b^	89.07	82.06	–	–	0.180	0.055	0.401	–	–	–
28_5K50K	89.94	84.01	89.80	83.27	0.250	0.100	0.427	0.200	0.050	0.384
29_5K50K	96.24	93.12	87.55	79.76	0.283	0.085	0.426	0.201	0.075	0.387
30_5K50K	87.89	81.23	88.32	80.55	0.215	0.100	0.535	0.162	0.061	0.310
31_5K50K	90.16	82.17	65.05	41.57	0.243	0.109	0.413	0.03	0.001	0.260

^a^Genotype imputation was from 5K to 50K using two types of reference population: (i) fixed reference population containing a large number of animals from all breeds and (ii) within-group reference population

^b^Scenario defined for the calculation of SNP r^2^ using 1000 animals as imputed

^c^CRAll and r^2^All = concordance rate and squared Pearson correlation, respectively, using the FIMPUTE software when a large set of animals from all breeds was defined as the reference population

^d^CRW and r^2^W = concordance rate and squared Pearson correlation, respectively, using the FIMPUTE software when the within-group population was defined as the reference population

^e^MeanA, MinA, MaxA, MeanW, MinW and MaxW = mean, min and max relationship among the 10 most related animals between the reference and imputed sets (all animals (A) or within-group (W))

The highest gain (25.11 % in CR and 40.6 % in r^2^) was obtained for Scenario 31_5K50K for which the reference population of 138 animals (within-group reference set) that was used to impute composite animals was replaced by a larger set consisting of 15,443 animals (see Table 6, fixed reference population for all scenarios). Figure 5 shows imputation accuracies per animal for imputation from 5K to 50K with a reference population composed of animals from the same group as those to be imputed (within-breed imputation): they are sorted from the highest to the lowest CR accuracy.

Imputation of Romney animals with different breed proportions (<100 % and <65 %), Coopworth (<70 %), and of composite animals, benefited from using a unique large reference population that included animals from all breeds/groups. Imputation of animals 100 % Coopworth and Perendale did not benefit substantially by including animals from all breeds/groups in the reference population compared to a within-breed reference population, with only a slight change in imputation accuracy observed for a few animals (see Fig. 5, Scenarios 28 and 30).

### Comparison of BEAGLE and FIMPUTE

Accuracies of imputation and corresponding computing times for FIMPUTE and BEAGLE are provided in Table 4. FIMPUTE outperformed BEAGLE across all scenarios. Overall, average decreases in accuracy of 3.06 % (CR) and 4.59 % (r^2^) for imputation from 5K to 50K and of 3.42 % (CR) and (r^2^) for imputation from 5K to HD were found with BEAGLE compared to FIMPUTE. Computation time was shortest in Scenario 1_5K50K for both software packages: 57 s with FIMPUTE and over 2 h with BEAGLE. Twenty GB of RAM (random-access memory) were allocated for both algorithms. For some analyses that failed using BEAGLE, the RAM threshold had to be increased to 100 GB for the computation of scenarios that investigated imputation to HD genotypes. Scenarios that used BEAGLE and were not completed within 5 days or failed twice are not presented in this paper and the cause of these failures was not determined. Imputation with BEAGLE in all Scenarios from 7_5K50K to 31_5K50K (results are only presented for FIMPUTE in Tables 5, 6 and 7) was not feasible and is not reported here.

**Table 7:** Rare allele imputation accuracy (r^2^) for different ranges of MAF

MAF	Number of SNPs	r^2a^
0 < MAF = 0.0001	35	0
0.0001 < MAF = 0.001	96	6.6
0.001 < MAF = 0.01	625	38.9
0.01 < MAF = 0.05	2360	57.8

^a^Allelic imputation accuracy (r^2^) for Scenario 27_5K50K where 1000 Romney animals were imputed using a within-breed reference set that included 4256 animals

Table 5 shows the accuracy of genotype imputation from 5K to 50K that was reached with FIMPUTE for Scenarios 7_5K50K to 24_5K50K.

### Predicting imputation accuracy before imputation and relatedness

Figure 6 shows imputation accuracy per animal across two scenarios measured by concordance rate (CR) according to the average number of Mendelian inconsistencies (MI) observed with 5K (Fig. 6a) and 50K (Fig. 6b) panels: a similar trend is observed in both plots.

The highest imputation accuracy (98.7 % in CR) was obtained for an individual for which the average MI between itself and the top 10 most related animals in the reference population was equal to 176.9 when the 5K panel was used and 1208.7 when the 50K panel was used. The lowest imputation accuracy was found for an animal for which MI was equal to 504.2 and 3297.9 when the 5K and 50K panels were used, respectively.

Tables 4, 5 and 6 also show the top 10 relationships between animals from the imputed and reference populations. The mean, minimum and maximum average top 10 values across all scenarios were equal to 0.129, 0.041 and 0.296, respectively. Scenario 15_5K50K (imputation of Romney animals using the Primera group as the reference population) resulted in the lowest values of relatedness [0.004 (mean), 0.003 (min) and 0.006 (max)]. Imputation of Coopworth animals using all the other animals as the reference population resulted in the highest average relatedness value (0.283) and in one of the highest imputation accuracies (CR = 96.24 %). After carefully examining the classes of relationship among the individuals in the reference population and imputed set (results not shown), we found that, in most cases, the most highly related animal was a half-sib, with genetic relatedness dropping quickly, where the relationship for the 10th animal in the top 10 most related set was close to 0.03 (Min Top10 stats in Tables 4 and 5). This indicates that in the scenarios that were designed for this study, the number of highly related animals (for example, family members that are shared between imputed and reference sets) was quite small. This is confirmed by the comparison of Scenario 1_5K50K (Table 5) with Scenario 3_5K50K, for which the reference population was enlarged by the addition of 31 animals that were highly related with animals in the imputed set; in this case the Max Top10 statistics did not exceed 0.234.

### Imputation of chromosome tails and rare alleles

Figure 7a shows imputation accuracies (r^2^) per SNP for the 26 autosomal sheep chromosomes for the animals described in Scenario 27_5K50K, in which 1000 animals were used as the imputed set. In general, imputation accuracies for the SNPs that were located at each end of each chromosome were lower than for those in other chromosomal regions. Figure 7b shows that 14 out of the 26 autosomes had at least one of their extremities covered by 100 SNPs with an average imputation accuracy lower than 40 % (r^2^).

Chromosome 4 shows the best marker coverage at the proximal end (average r^2^ = 80.40 %) whereas the lowest imputation accuracy was found for the 100 SNPs located at the telomeric end of chromosome 1 (average r^2^ = 29.89 %).

Imputation accuracies of rare alleles as measured by r^2^ and grouped into four categories according to the MAF of each imputed SNP allele (0 < MAF < 0.05) are in Table 7. Thirty-five SNPs were reported in the first category (0 < MAF < 0.0001) and their r^2^ was equal to 0. The overall average imputation accuracies (r^2^) for the MAF groups (0.0001 < MAF < 0.001; 0.001 < MAF < 0.01; and 0.01 < MAF = 0.05) were equal to 6.6, 38.9 and 57.8 %, respectively.

### Genetic relationships among breeds based on MDS cluster

**Fig. 8:**
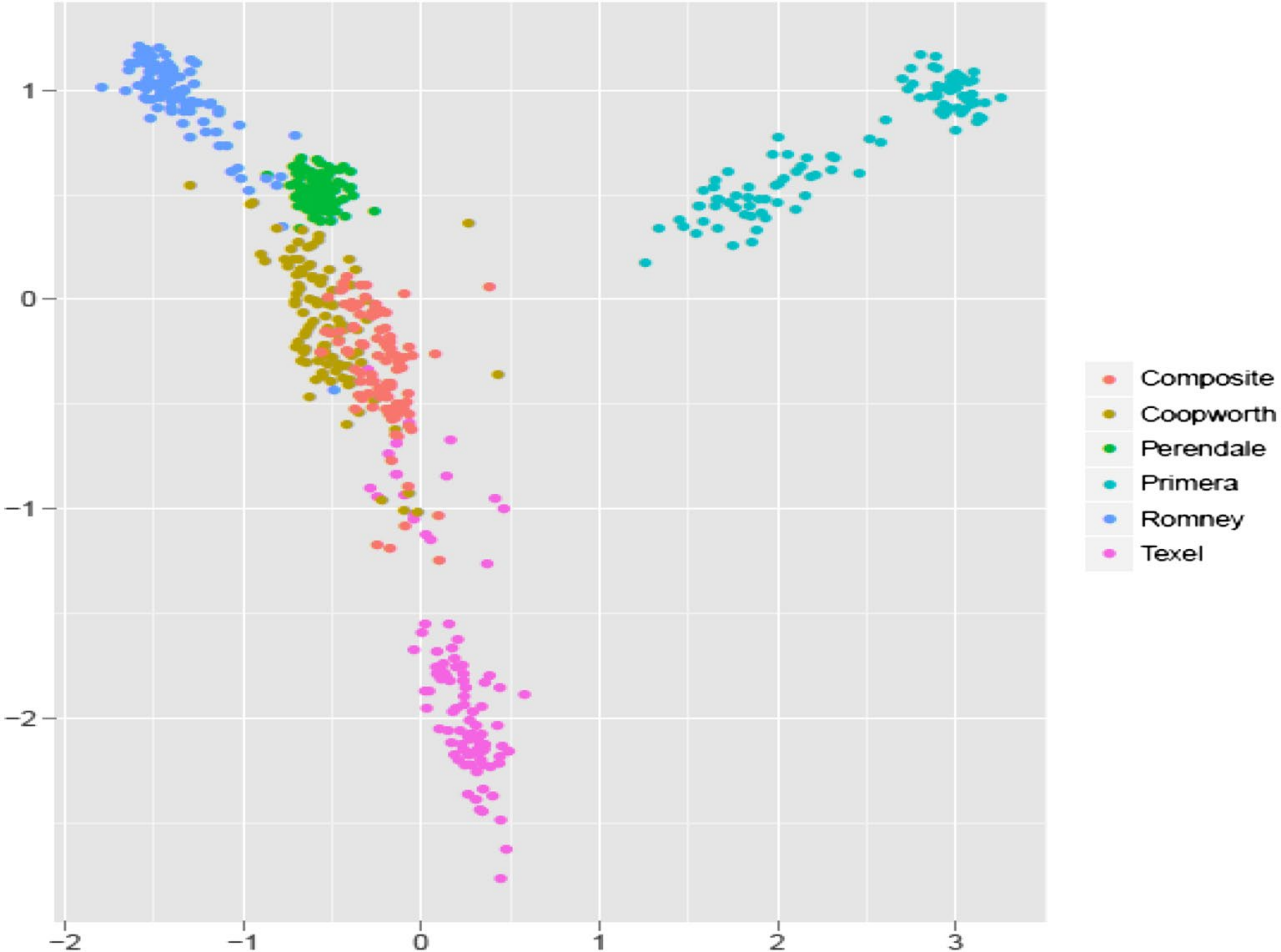
MDS Cluster plot illustrating the genetic relationship (based on the genomic distances obtained by SNPs) between animals of each group or breed used to describe the genetic structure of different groups/breeds and to better define the imputation scenarios

 Figure 8 illustrates the genetic relationships (based on genomic distances estimated from SNPs) between animals of each group or breed. Primera and Texel groups showed reduced connectivity with other breeds (Romney, Coopworth, composites and Perendale). This plot was used to determine the most relevant imputation scenarios and for the description of population structure.

## Discussion

We used a 50K SNP subset that was extracted from the HD panel to compare the imputation accuracy from 5K to 50K, 5K to HD using a one- or two-step procedure, and from 50K to HD. Animals genotyped with the HD panel were not re-genotyped with the 50K panel, but the 50K panel was derived as a subset of the HD genotypes. The large number of animals (17,176) that were genotyped with the Illumina OvineSNP50 (50K) panel allowed us to investigate the use of alternate reference populations, i.e. that comprised samples of animals of various sizes and breed composition, the impact of removing animals that were closely related to the reference population and also to identify the chromosomal regions that are not imputed efficiently in Romney animals, for which a large imputed set (N = 1000) was used to reduce the bias in r^2^ imputation results.

### Impact of reference population on the imputation of purebred and crossbred animals

Imputation accuracies that were obtained in our study were on average higher than those reported by Hayes et al. [14] for Australian sheep. These authors investigated different breeds and smaller populations. Imputation accuracy depends on several factors, including the number of immediate ancestors in the reference population, size of reference population and density of the SNP panel used for both imputed and reference sets [13]. Scenarios 7 to 11 in our study resulted in a substantial gain in accuracy by enlarging the reference population used for the within-group imputation of Romney animals which agrees with the findings of [13]. Ventura et al. [1] investigated the accuracy of imputation from 5K to 50K in a multi-breed beef cattle population and reported higher CR accuracies when closely-related individuals to the imputed group along with a representation of the breed composition of the imputed group were included in the reference population. These authors also showed that adding another purebred population in the reference population did not improve the within-breed imputation for imputation from low- to medium-density panels. Sargolzaei et al. [9] reported that imputation from denser panels (i.e. from 50K to HD) depended less on the size of the reference population than that from sparser panels (i.e. from 5K to 50K). The existence of strong relationships between animals in the reference and imputed sets, helps to better detect long haplotypes that are used to infer missing SNPs. Hayes et al. [14] cited problems of pedigree structure and small family sizes in sheep breeds, which affect the imputation process if a population imputation method is not applied. McRae et al. [29] reported that, in sheep, the linkage disequilibrium between SNPs that are separated by less than 10 cM is lower than that for SNPs separated by similar distances in the dairy cattle population, thus reducing the power of the population imputation method which depends on linkage disequilibrium. The number of haplotypes shared between breeds is small and a large reference population is required to capture haplotype diversity for different sheep breeds [1]. Imputation accuracies were higher for almost all the scenarios for which a fixed and large reference population was used and this was consistent with the above studies. Across all scenarios (FIMPUTE was the only software used) for imputation from 5K to 50K, a slight loss in accuracy when using the fixed and large reference population was observed only for a few animals. In addition, a large gain in accuracy for a large proportion of animals (purebred and crossbred) in the imputed set, justifies the use of a fixed and large reference population for all situations. This may be associated with the complexity of the breed composition of each animal considered in some cases as purebred.

The top 10 measures of relatedness demonstrated that accuracy of imputation was strongly associated with the level of relationships between animals in the imputed and reference sets and that it increased as the average top 10 relationships increased. The relationship between imputation accuracy and top relationships was also demonstrated by Bolormaa et al. [22].

### Imputation from 5K to both 50K (HD subset) and HD panels

Imputation from 5K to HD was slightly better (0.6 %) than imputation from 5K to 50K. This result is not consistent with previous studies in other species. A study on Hereford cattle by Picolli et al. [8] showed that imputation accuracies were higher for imputation from 5K to 50K (CR = 94.60 %) than for imputation to HD (CR = 89.80 %). This implies that longer chromosome segments need to be inferred if the targeted SNP density for imputation is the HD panel, when the number of SNPs in the low-density panel is fixed (5K). The fact that there are more misplaced SNPs in the medium-density panel (50K), compared to the HD panel may cause more problems when imputing to 50K from the same low-density panel. Further studies with other datasets are necessary to check this issue. Imputation of animals that are highly-related to individuals in the reference population can benefit from the identification of long haplotype blocks and thus could lead to smaller differences in imputation accuracies for imputation to 50K and HD panels from the same low-density panel. The difference in overall imputation accuracy between imputations to these two panels is reduced from less than 1 to 0.2 % if animals with lower CR than 80 % are not considered in the statistics (animals that are weakly related to those in the reference population). Further investigations on this topic are also necessary. Individuals for which the 5K SNPs were imputed to 50K with an imputation accuracy lower than 70 % (Fig. 2a, b) had an overall average gain in CR accuracy of 21.32 % after imputation from 50K to HD panel. According to Sargolzaei et al. [9], closely-related animals share long haplotypes that usually occur at a low frequency in the population, while less related individuals may share short haplotypes that occur at higher frequencies in the population. Based on these results, it is likely that these short haplotypes were captured by increasing both panel densities (i.e. imputation from 50K to HD compared to 5 to 50K) and the effect was largest for the animals for which imputation accuracies were lowest in the imputation using the low-density panels.

### Imputation from low- and medium-density panels to the HD panel

The two-step imputation from 5K to HD (5K to 50K and then from 50K to HD) outperformed the one-step imputation from 5K to HD (+5.67 % in CR). A comparison of one- and two-step imputation approaches in Canadian dairy breeds (Ayrshire, Guernsey and Holstein) reported by Larmer et al. [10], also showed that the two-step procedure resulted in higher accuracies. A similar study on Braford and Hereford beef cattle in Brazil [8] reported a gain in CR of 8.06 % with a two-step imputation procedure. These authors suggested that the gain in accuracy can be attributed to the larger number of SNPs present in the low-density (50K) panel used in the second step of imputation.

### Imputation of rare variants

Imputation of rare variants was recently investigated on human data [30, 31]. Kreiner-Møller et al. [32] proposed a new approach to improve imputation accuracy of rare alleles that was based on a two-step imputation procedure, i.e. (step 1) genotyping many additional individuals only for the rare variants to constitute a specific reference population for the rare segments and (step 2) imputation to the highest density panel as usual. Using data on a purebred dairy cattle population, Sargolzaei et al. [9] showed the importance of having information on closely-related animals for the efficiency of the imputation of rare variants and reported gains in accuracy relative to the increase in reference population size and panel density [9]. These authors showed that rare variants tend to be recent events and are directly associated with longer haplotypes. They reported imputation accuracies for rare variants using various sizes of reference populations and found that they were higher than 80 % for a reference population size similar to that described in this study (N > 4000). This pronounced disparity in imputation accuracies between our study (58 %) and the study of Sargolzaei et al. [9] in dairy cattle (at least 80 %) is mainly due to differences between the structure of dairy and sheep populations. Population structure will also directly affect the number of closely-related animals that will positively influence the imputation of rare variants. The imputation accuracies (r^2^) of 0 (N = 35) for SNPs with MAF lower than 0.0001 that were obtained in our study are likely due to genotyping errors or the absence of variation for this specific set of SNPs, which directly impacts the correlation calculation.

### Software comparison

We chose the version 3.3.2 of BEAGLE for our study because it is implemented in practice for genomic selection in New Zealand sheep at the industry level [20]. Computation run-time and efficiency of BEAGLE and FIMPUTE software packages have been reported by several authors in other species [1, 8, 9]. Our results corroborate the findings from those authors and show that FIMPUTE V2.2 outperformed BEAGLE 3.3.2 across all imputation scenarios. Since FIMPUTE is able to parallelize chromosomes on multi-core systems [9], it will become an important tool for imputation of thousands of animals genotyped with a variety of panel densities.

### r^2^ and concordance rate measures of imputation accuracy

Concordance rates as a measure of imputation accuracy have been reported by several authors, including for imputation in sheep [14]. Sargolzaei et al. [9] used allelic r^2^ (squared correlation between imputed and true genotypes) as a measure of imputation accuracy that minimizes the dependency of SNP allele frequencies. The r^2^ calculation can be carried out on a SNP or animal basis. Unlike the calculation on an animal basis that uses the large number of SNP genotypes per animal for the calculation of r^2^, the calculation of r^2^ per SNP requires a large number of animals to compose the imputed set, in order to obtain an unbiased estimate of the correlation. For this reason, Scenario 27_5K50K was considered the most appropriate (for which the imputed set included 1000 animals) to estimate r^2^ accuracy per SNP. Our study used three different measures of imputation accuracy depending on the scenarios: concordance rate (plots reporting imputation accuracy per individual in Figs. 2, 3, 4, 5 and 6, reported as an average value in Tables 4, 5 and 6) and r^2^, both per animal (Tables 4, 5 and 6), and per SNP (Scenario 27_5K50K), used to investigate regions that were imputed less accurately.

### Prediction of imputation accuracy before imputation

Imputation accuracy can be determined only after masking chromosome segments from the individual’s genotype and by comparing the true and masked genotypes to the imputed genotype. According to Calus et al. [13], imputation accuracy depends mainly on the ability of identifying the correct haplotype for a specific SNP and on the number of genotyped immediate ancestors. In this paper, we report a novel and efficient approach to identify, prior to imputation, the animals for which regions in the genome are less likely to be inferred efficiently. Imputation from 5K to 50K and HD SNP panels was investigated and we found that there was a clear trend relating the resulting imputation accuracy with the number of MI at the 5K genotype level (before imputing). The same trend was observed using the 50K genotypes (original and not masked genotypes). MI values (average value between an imputed animal and the top 10 related individuals from the reference group) higher than 400 (measured at the 5K level) or 3000 (at the 50K level) were obtained for individuals for which imputation accuracy was lower than 80 %. Further analyses are necessary on other populations with a different structure to better evaluate this method. If the imputation process is evaluated for denser or sparser panels, a similar investigation with different SNP densities is required.

### Imputation efficiency per chromosome region in Romney animals

According to Picolli et al. [8], in beef cattle, imputation accuracy is associated with chromosome length. They reported that CR accuracies were highest for bovine chromosome 1 and lowest for chromosome 28, which is consistent with our results. However, little is known on the imputation accuracy of proximal and telomeric regions for each chromosome in sheep. We showed that only ovine chromosome 26 had an overall imputation accuracy over 100 SNPs at each end higher than 60 % (r^2^). Most of the ovine chromosomes had problems at least at one of the ends. If a trait is affected by a locus located in one of these regions, association studies will be impacted or biased if the genotypes investigated are imputed. Incorporation of additional SNPs located in these regions in the low-density panel may improve imputation accuracy.

## Conclusions

In this study, we identified several critical factors that influence imputation accuracy and that need to be taken into account for the implementation of genomic selection in industry breeding programs for New Zealand dual-purpose sheep breeds. These factors include the SNP panels and software used, both of which should be carefully evaluated when new technologies are presented. Strategies of imputation (one- or two-step) and the choice of the animals to be genotyped using both high- and low-density panels are important since we highlighted the influence of the presence of closely-related animals in the reference population as well as the improved imputation accuracy reached when a subset of more closely-related animals is added to the reference population compared to a larger reference population that includes all the animals. Incorporation of additional SNPs in the lowest density panel (5K) increases imputation accuracy furthermore. Since it is not possible to have a high imputation accuracy for all the animals, we present a method that allows imputation accuracy to be predicted based on the low-density genotypes, which can then be used to restrict genomic prediction only to animals that can be imputed with sufficient accuracy. Imputation of rare alleles is a difficult task that needs to be better investigated in future studies, especially for regions under selection pressure and for scenarios for which the size of the reference set is limited.
